# Place branding: Religion in shaping the three-dimensional essence of a city brand through stakeholder engagement

**DOI:** 10.1371/journal.pone.0296162

**Published:** 2024-01-23

**Authors:** Xiufang (Leah) Li, Abdullah Alahmari, Bruno Schivinski

**Affiliations:** 1 School of Media and Communication, RMIT University, Melbourne, Australia; 2 Department of Media and Communication science, Islamic University of Madinah, Madinah, Saudi Arabia; St John’s University, UNITED STATES

## Abstract

This study explores the role of religion in engaging stakeholders in branding a place on social media and unmasks what implications this has for (re)constructing the three-dimensional meanings of a place brand. Using the content analysis method to examine the case of Saudi Arabia, it probes how the key stakeholder groups of the government and the residents structure and interact with the narratives of the cities—Jeddah and Riyadh—on Twitter, Facebook, and Instagram. The results show the Islamic religion serves as a powerful tool for motivating the residents to engage in the government-led city branding initiatives at the individual level. However, the strategy of dwelling on religion to mobilize resident engagement at the individual level towards the social level with the aim of growing resources in support of social development should be reassessed within a dynamic social system. Theoretically, the proposed framework of religion city branding expands the scope of stakeholder engagement in place branding research through the integration with the driver of religion, especially unveiling how religious factors shape the personality traits of a place brand. It contributes to the practical sense that religious elements might be deployed by the key stakeholder groups of the government and residents in city branding initiatives, which potentially contributes to their relationship and the engagement of residents in co-creating a place brand with the government. This Saudi-focused study, therefore, possesses significance for place branding practices in Middle Eastern countries and beyond.

## 1. Introduction

The consideration of religion in branding a city in the Middle Eastern region is critical. Marketers are conscious of the massive inclination of the public of the respective cities to emphasise the religious implications for their spending habits [1]. Cities, which are mostly dominated by the Muslim community, have manifested authoritativeness in defining their interests, as witnessed by the boycott of products that defy Islam [2]. Therefore, a strong moral and spiritual obligation characterizes the ways in which a city located in the Middle East is branded.

Cities operate within diverse and complex environments of social interactions, which affects connectivity and spiritual well-being [3]. The personality and cognitive growth of the city can create a logical position, which can also be viewed from a religious perspective [4]. City branding is all about creating a vision that is compatible with the city’s identity and local circumstances [5]. It is perceived to be identity-driven, aiming to convey the unique principles and narratives of the site [6]. Cities are located in a diverse range of geographical locations, and how city inhabitants form their personal links with city labels may vary [5]. From this perspective, the starting point for any city branding needs to rest on the pre-existing cultural, economic and natural qualities that organically form the place’s identity [5].

Religion is regarded an essential form of culture and it is widely agreed that there are a multitude of measures of religious facets [7]. Religious orientation can influence trustworthiness [8], the credibility of a city brand [9], and the loyalty of residents to this place [10]. Religion as a multidimensional construct can be measured by a set of key religious factors, including religious beliefs (e.g, concepts/principles of God, supernatural agents), rituals (e.g, the *hadj* pilgrimage in Islam, daily meditation in Buddhism), values (e.g., segregation in Islam, forgiveness, purity/sanctity), and community (e.g., a sense of belonging, group identification, collective self-esteem, that serves to create social bonds between religious members) [7]. An individual’s level of religiosity regarding these four religious constructs has the influence on their attitudes and behaviors towards a brand, including level of loyalty to this brand [7,11]. Religious residents might appreciate a city brand that upholds their individual religious beliefs. The residents’ personal values, well-being, and motivations are essential in determining the destination image and the personality traits that a specific city is trying to create [12].

Stakeholder engagement is identified as a method of mobilizing participation in a given environment during the processes of place branding. Residents respond positively to government-led branding campaigns provided their needs are expressed and met; and the stakeholder groups of the government and residents are the key actors in place branding initiatives [13]. Successful place branding campaigns drive behavioural engagement with the city [14]. In the context of the Middle East, this implies that government-led branding initiatives need to resonate with the values of the residents of this place, and one of these values is their religious beliefs. The role of religion in branding is an indication of prioritising engagement, which allows for the production of long-term as opposed to short-term benefits for a place [15]. This form of resident engagement with city branding has ensured that the local government is no longer the sole source for communicating a city brand [16]. From this perspective, the practice of stakeholder engagement as a guide to developing and implementing city branding strategies is vital to ensure that the values of residents are incorporated into city branding exercises.

In Saudi Arabia, city branding involves meeting the expectations of a deeply religious community as guided by stakeholder engagement. This trend is in the interest of a diverse range of stakeholders from the aspects of achieving business success and creating social impact. Existing research concentrates on examining how governments leverage religious architecture in branding cities [17], which involves the physical attributes of a city brand. However, it is important to engage all of the stakeholder groups, especially the residents, in co-developing a city brand through the three dimensions of a place brand, including physical attributes, functional attributes, and personality traits [13,18]. Hence, this article attempts to investigate the role of religion in engaging stakeholders on social media in branding a place and unpack what implications this has in the (re)construction of the three-dimensional meanings of a place brand. To achieve this research aim, this article addresses the following research questions (RQs), including *how government actors use religious factors to engage residents in branding Saudi Arabian cities on social media (RQ1)*? *How the residents respond to the government’s attempt on social media (RQ2)*? and *what the implications for empowering residents in city branding by using religion are (RQ3)*?

Using the content analysis method, we focus on analysing the networked narratives produced by the Saudi government and the residents surrounding the Saudi Seasons on social media in response to RQ1 and RQ2. We then discuss the implications of that for city branding resting on religious factors in the last section “Discussion and conclusion”, addressing RQ3. The research outcomes of this article enrich the existing literature of stakeholder engagement in place branding by integrating religious factors into place branding research and unveiling how these factors influence the three-dimensional essence of a city brand, especially the brand’s personality traits. Practically, it contributes to the sense of how religious factors are expressed and (re)structured by the key stakeholder groups of residents and the government in city branding initiatives, which might potentially contribute to their relationships and the engagement of residents in co-creating a city brand with the government. This study therefore advances the relational approach in stakeholder engagement through leveraging religion, which potentially empowers religious communities in the promotion of a place.

## 2. Literature review

### 2.1. City branding and the three dimensions

A place brand comprises the following three dimensions, including personality traits, functional attributes, and physical attributes [18]. Physical attributes are a place brand’s distinguishing characteristics, which might include the name, word, logo, symbols, style, design, or any combination of these characteristics [18]. When physical attributes such as city architecture and city logos elicit personal resonance, places become meaningful. Functional attributes denote the tangible benefits that a place provides its inhabitants with [18]. These functional attributes can take various forms, such as a clean environment, business opportunities, innovative businesses, retail outlets, cafes, transport, cultural activities and government services [19]. Personality traits are linked to intangible meanings of a place brand, such as the brand’s emotional and cultural components [20], as well as its connection to citizens’ beliefs and ambitions [18]. The dimension of personality traits in city branding, thus, comprises social bonding, city brand attitudes (towards the lifestyle and reputation of the city where a resident resides in), and intentions (of continuing to live or retire in the current city) [19,21], in addition to the aforementioned religious factors within a religious society. These three dimensions are interdependent, meaning that appealing personality traits of a city help to cultivate a positive relationship between the place and its target public, and this in return is likely to motivate the target public to pursue the products or services offered by this place [9].

City branding is not only a process of negotiation between places and residents [22], but also the exercise of interactions between local governments and residents [23]. From the government’s perspective, city branding aims to develop and communicate a city’s identity to gain public support for domestic political structures and governance [24,25]. Therefore, social participation, described as concerted action achieved by a diverse set of partners situated within the context of a socially complicated environment [26], is a potent tool for achieving this objective during city branding processes [23].

### Stakeholder engagement on social media

#### Individual-level engagement and social-level engagement

The concept of engagement has emerged to describe the involvement of stakeholders in a brand through cognition, emotion, and behaviour [27]. This concept is extended to the area of place branding. Individual-level engagement refers to an individual’s involvement in place branding initiatives, which could enhance the sense of belonging among the residents and contribute to the co-creation of a place brand [13]. It focuses on creating desirable outcomes at an individual level through an individual’s active involvement in co-developing the three aspects of a city brand, including physical attributes, functional attributes, and personality traits [13], the outcome of which helps to shape the city’s identity. The conceptualisation of engagement is broadened from the individual to the social level. In a dynamic socially situated system, social engagement is characterized by collective action and outcomes among various stakeholders, formed by the basis of individual-level engagements [26]. The social level of engagement becomes a powerful instrument for the growth of resources to support the social development of a place, which is a contributor to raise the place’s competitive advantage and the status, that benefits a wide range of stakeholder groups [13,28].

#### Stakeholder engagement on social media

Stakeholder engagement is viewed as a strategic framework that can be used as a guide to evoke genuine interactions and conversations with different stakeholder groups in place branding processes [13]. It plays an important role in building a place’s identity which values collaborations among public, private and civic groups [29]. Therefore, the government of a city cannot brand places and build its reputation on its own due to lacking resources, including public support [30]. The government relies on a wide range of stakeholders, including private organizations, social groups, residents and visitors, to brand a place [30]. These stakeholders can drive a place’s status by reflecting on their collective attitudes, counteracting rivalry, offering intuitive feedback, and delivering appropriate value to the target audiences [29]. The interplay between public, private, and civil society groups shapes the identity of a place [31]. Within this diverse range of stakeholders, city residents and visitors are vital in shaping the reality of a place. They act as ambassadors for a place brand and serve as citizens and voters who obtain the right and obligation to participate in political decisions of the place [13].

Stakeholder engagement in city branding is facilitated by intricate and multidimensional communication on social media [32]. Stakeholder engagement should be undertaken in an authentic way, meaning moral, inclusive, and cooperative communication practice with a focus on engagement with residents [13]. Social media provides stakeholders with an environment to communicate, contribute, and share information. This environment enables a distinctive communication style—many-to-many rather than either one-to-many or one-to-one [33], which allows stakeholders to build and promote a place brand and reputation through the exchange of user-generated content [31]. Hence, stakeholders on social media construct different facets of a place brand in a variety of ways, which may be indirect or direct, intentional or unintentional [34].

Local residents adopt social media tools to influence the target group as the place’s co-creators or brand ambassadors [35]. They promote a place brand by sharing images on social media and creating temporal place values [36]. Their use of social media networks to communicate shared attitudes has an impact on the place’s reputation [31]. In response, governments around the world have expanded the use of new platforms to market a city and win public confidence [37]. The prominence of consumer content, viral marketing and searching requires governments to redefine their communication strategies that align with the trends of the digital environment [38]. For instance, the launch of applications endorses and encourages governmental openness and accessibility [38,39].

### Using religion to brand Saudi cities

City branding practices in Saudi Arabia show strong connections with religious rites and ceremonies. Saudi Arabia is one of the most religious places that the world, long known to Muslims. The examination of religion enables us to comprehend the influence that shapes the towns and cities of Saudi Arabia [40]. Religion serves as an external form of social function driving the society of Saudi Arabia [41]. Religious, tribal, clan-based social institutions and long-standing cultural standards have an authoritative influence on society [42]. This has an impact on the residents living in Saudi Arabia across cultural, economic, political, and social dimensions [40]. However, the advancement of social media has created changes in how residents engage with the government to express their needs in the place where they reside. This creates implications for the Saudi government when it brands Saudi Cities.

In Saudi Arabia, the teachings of Islam and the Qur’an and the Hadith–sayings—of the Prophet Mohammed (peace be upon him) guide every aspect of the daily life of all Muslims [43]. From this aspect, the incorporation of religion into city branding efforts helps to shape the physical attributes, functional attributes, and personality traits of a city brand. The physical attributes of a city brand in the Islamic religion are embodied in prayer facilities like mosques, colours that express the history of Islamic civilization, and architectural styles featuring Islamic heritage. A Saudi resident’s religious background determines how far they would live from the Mosque, and this affects housing design in the country [43]. Traditional neighbourhoods are centered around an enormous central Mosque that serves most of the city’s needs from residents to the government and commercial actors [43]. As a result, the physical attributes of a Saudi city contribute to the sustainability of the housing needs of the unique conservative Islamic culture of the Saudi residents [43].

The functional attributes of city branding practices from the perspective of the Islamic religion are demonstrated through the city’s support of the services that enable residents to live better while contributing to local governance. For instance, a clean and safe environment (e.g., public cleaning and natural care of plants which Islam supports), business opportunities, cultural activities, government services, transport, and political cohesiveness manifest the functional attributes of city branding practices from the perspective of the Islamic religion. The Saudi government leverages the power of law from Islam to punish residents who break the rule of maintaining a clean environment in Saudi cities [44]. Religious and long-standing cultural standards have an authoritative influence on businesses that are expected to comply with Islamic Law in their operations in Saudi Arabia [42]. Recognizing the importance of faith in bolstering the Saudi royal family’s political clout and cohesiveness, the Saudi ruling family views its organizations as an ambassador to ensure the best aspects of the Islamic religion [45].

The success of building the dimension of functional attributes facilitates the Saudi government’s quest to promote the cities as spiritual development centers [46]. The Saudi government emphasises spiritual and human values among residents in the city through social bonding, cultural diversity, and a positive attitude to multicultural society and community spirit [47]. It focuses on young residents who show unique cultural orientations compared to the older population [47]. With the increasing number of young residents using social media to spread the word about their faith [48], the trend of increased sensitisation of identity and choice has been brought to the front [49], and this has contributed to the generation of a universal culture [50]. As a result, Saudi residents are less committed to embracing local cultures and ideologies but diversity. Therefore, the personality traits of a city brand in Saudi Arabia are characterized by social bonding, diversity, and a positive attitude towards multicultural society and community spirit.

**Fig 1:**
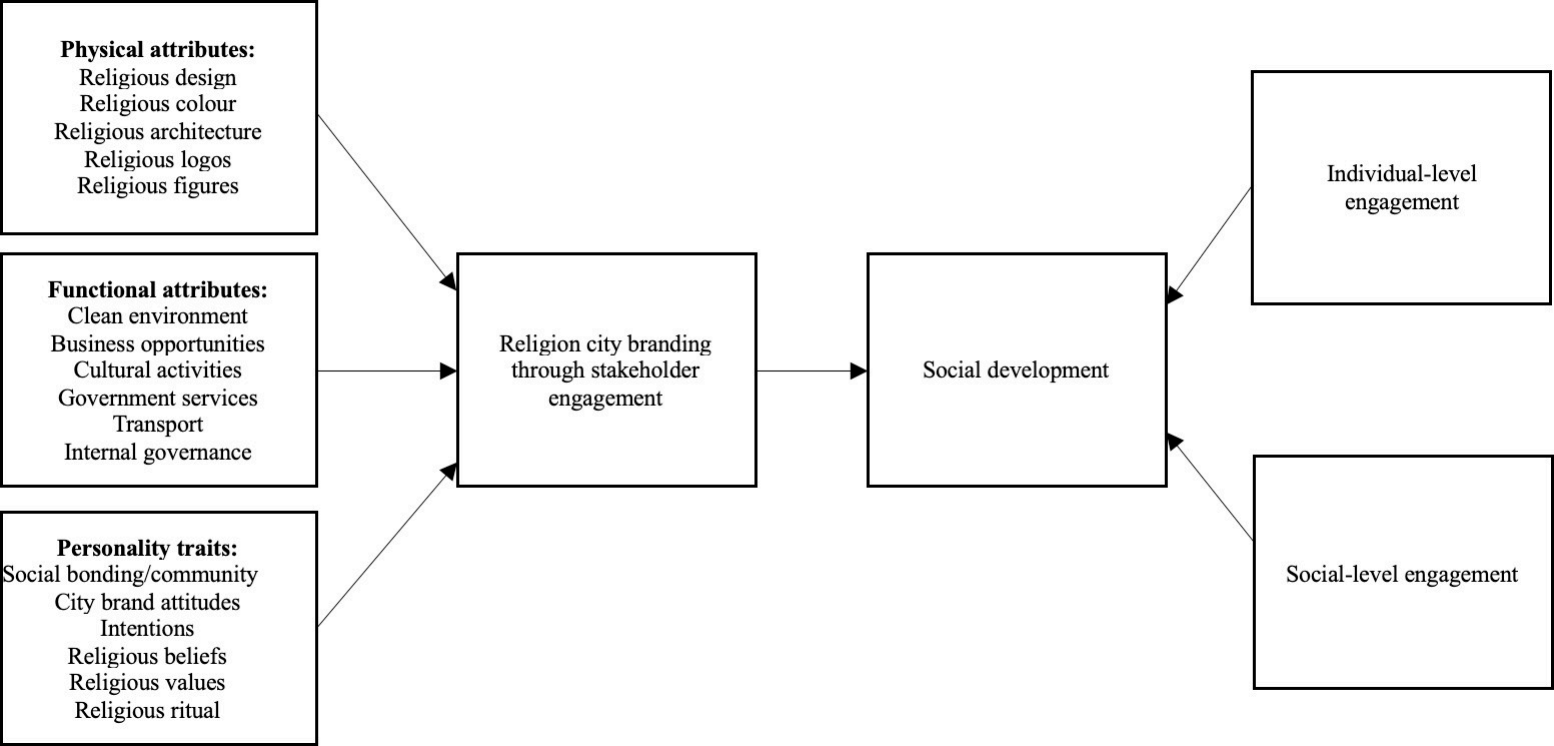
Framework of religion city branding through stakeholder engagement.

The discussions show the role of religion in evoking the dynamics of city branding practices via stakeholder engagement on social media, specific to the engagement between the government and the residents in Saudi Arabia. On the one hand, driven by potential rewards to economic growth and sustainable governance accounting for the mediation of religion, the Saudi government is inclined to initiate urban development in city branding exercises, featuring sustainability, technologies, and lifestyles. This, in return, provides the residents with access to live a better life. On the other hand, Saudi residents expect the government to raise the quality of life by enhancing the three dimensions of a city brand, including physical attributes, functional attributes, and personality traits. Drawing upon the principles of stakeholder engagement in place branding, the accomplishment of these goals requires the government to engage the residents in co-developing the three-dimensional essence of a city brand at the individual level. The success of this individual-level engagement in city branding initiatives lays the foundation for generating social-level engagement that contributes to the resources of social development in a religious society. Therefore, we propose the framework of religion city branding through stakeholder engagement. See Fig 1.

## Methodology

### 3.1. The two Saudi Seasons

The Saudi Ministry for Tourism and National Heritage has been celebrating the Saudi Seasons, known as Saudi Festivals, annually since 2019. This initiative is led and planned by different inter-related Saudi authorities, including the Ministry of Culture, the General Authority for Entertainment, the Ministry of Sports and the Saudi Exhibition and Convention Bureau, under the leadership of a committee led by the Crown Prince Mohammed bin Salman. In this study, we chose two Saudi seasons, the Jeddah Season of 2019, and the Riyadh Season of 2019, to analyse the cities of Jeddah and Riyadh, respectively, in Saudi Arabia.

First, Riyadh is the capital city of the Kingdom of Saudi Arabia. With a population of more than six million, Riyadh is a point of origin of Saudi Arabia characterized by historical depth, and possesses political and commercial power in the region [51]. The magnitude of this development in the city’s infrastructure will fundamentally modify the nature of the urban area [51]. It will also assist Riyadh in emerging more obviously as a unique city brand instead of merely being known as the capital city of Saudi Arabia [51]. Therefore, the Riyadh Season would change the image of Riyadh city. Second, Jeddah city is a stopover destination for the Islamic cities of Makkah and Medina on the Red Sea. Due to the influx of Muslims from all over the world on pilgrimage, Jeddah has become one of the most culturally diverse cities in the world, and Jeddah Season offers a great opportunity to showcase this diversity. Therefore, the Saudi government created the Saudi Seasons as a strategic plan for developing Saudi cities to be one of the top 100 cities to live in the world.

### Content analysis

This study used a case study approach to examine the networked narratives produced by the Saudi government and residents during the Saudi Seasons on Twitter, Facebook, and Instagram, specific to RQ1 and RQ2 through using the content analysis method. The method of content analysis is very helpful to examine social media content [52], as it yields a systematic and objective exploration of the content of published material [53]. The use of case study provides insight into a wide range of indicators, allowing governments and scholars to effectively evaluate engagement between stakeholders [54].

State and non-state actors distribute information and engage with their audiences on social media [55]. According to the social media statistics of the Kingdom of Saudi Arabia, more than 90% of the population use social media [56]. Social media platforms offer the venues that allow for high levels of communications with residents and tourists [57]. This explains why the use of social media has become one of the important components of city branding strategies. Therefore, this study collected social media data on Twitter, Facebook, and Instagram.

All posts were purchased from a professional service provider during the research timeframe. According to the recommendations of social media researchers [58,59], the researchers opted for the assistance of Podargos Technologies. The research period was set between June 08 and July 18, 2019 for the Jeddah Season, and between October 11 and December 30, 2019 for the Riyadh Season. This study has received the approval granted by the College Human Ethics Advisory Network (CHEAN) at RMIT University, as it meets the requirements of the National Statement on Ethical Conduct in Human Research. The posts collected in this study took the mode of texts rather than images, videos, and audios, including the texts sourced from the government accounts as well as the texts generated by the residents in their responses to these government posts. Coding social media content requires a coding scheme. As discussed, a city brand is demonstrated through physical attributes, functional attributes, and personality traits [13,18]. Therefore, aligning with the proposed framework of religion city branding, we developed the following coding scheme to examine RQ1 (see Table 1).

**Table 1:** Coding scheme: Brand narrative of the cities on social media.

*City season*	*Physical attributes*	*Functional attributes*	*Personality traits*
Jeddah / Riyadh	Religious design	Clean environment	Social bonding/community
	Religious colour	Business opportunities	City Brand Attitudes
	Religious architecture	Cultural activities	Intentions
	Religious logos	Government Services	Religious beliefs
	Religious figures	Transport	Religious values
		Internal governance	Religious ritual

The indicators of ‘like’, ‘share’ and ‘comment’ assess user engagement on social media [60]. Increased use of these interactions indicates an active relationship between social media users and the brands with which they interact [61]. It also suggests user engagement and interest in the contents of online posts as well as their willingness to establish a line of communication [62]. According to this metric, the three factors of popularity (P), virality (V) and commitment (C) are used to understand resident engagement on social media. Popularity (P) is defined as user messages’ appeal and reputation = [(Total number of likes/total number of posts) / (number of followers)] * 1,000; Virality (V) shows users’ interest in the city brand and its social media-shared content = [(Total number of posts shared/total number of posts)/(number of followers)]*1,000; Commitment (C) refers to producing additional online content, which represents a deeper level of interactions with fellow users and the city brand itself) = [(Total number of comments/total number of posts)/(number of followers)]*1,000; and engagement (E) is determined as the sum of these three factors, meaning E = P + V+ C [13,63]. This study utilized the above metric to measure the engagement of residents with the posts circulated by the local governments on the three social media platforms during the two Saudi Seasons, addressing RQ2.

Two coders with a week of training used the above coding instrument to manually code the data. They were native speakers of the Arabic language, and all posts were in Arabic. Each of the coders coded the data separately, and the researchers compared their coding results to examine the respective interpretations of the coders and recognise the points of significant divergence [64]. After the training was completed, the researchers used 25% of the posts (*n* = 313) as the reliability sample, and the reliability analysis was determined to be 0.86 using the Holsti’s formula [65]. A value of 0.80 or higher was considered dependable [65]. After that, the coders coded 1253 posts. Thus, the researchers analysed the posts in Arabic and the findings were translated into English. We then discussed the findings in relation to the implications for empowering residents in city branding leveraging religion in the last section “Discussion and conclusion”, responding to RQ3.

## Findings

RQ1 examined how government actors used religious factors to engage residents in branding Saudi Arabian cities on social media. According to the results, the government concentrated on developing narratives linked to the functional attributes of the Jeddah and the Riyadh city brands, evidenced by 666 posts accounting for 53% of the total posts. This was followed by the portrayal of the personality traits of the two city brands, seen through 393 posts—32% of the total posts. The government, however, paid little attention to portraying the physical attributes, supported by 194 posts—only 15% of the total posts. This result indicated that the Saudi government portrayed the two seasons as a religion-based cultural event in an attempt to appeal to the residents, which mainly conveyed the functional attributes of the two cities.

Specifically, the posts relating to the dimension of physical attributes revealed the religious incentives that the government relied on to make the two cities attractive. For instance, the government articulated the development of the infrastructures in these cities by illuminating the design of historic Islamic buildings in mosques. The mosques present Arabic calligraphy as a form of Islamic arts. Therefore, the aesthetic expression of Islamic architecture is communicated through the symbol of Arabic calligraphy, that is associated with the teachings embodied in the Holy Qur’an.

Regarding the posts structured into the dimension of functional attributes, the analysis showed the government addressed the residents’ demand for improving local amenities, including a clean environment, more cultural activities, and better transport and government services. For example, the Saudi government hosted cultural activities for the purpose of memorizing the Noble Qur’an, which is the holy book of Islam; and ran a competition for the most beautiful voice in reading the Qur’an. The Farouq competition as one of the Saudi government’s initiatives aims to revive the prophet Mohammed’s commandment: “Teach your children swimming, archery, and horse riding.” To encourage the people to participate in this competition, the government launched a total of over US$ 300.000 to reward the winners. The Saudi government further created the posts to promote outdoor and indoor recreations by illustrating the entertainment options provided by the community centers in the cities. In addition, it depicted how the road networks were developed and how the government resolved the problem of traffic congestion that prevented the local people from joining cultural events in the cities.

According to the posts involving the dimension of personality traits, the government made an attempt to engage the residents via highlighting social bonding/community, city brand attitudes, and religious beliefs. For an example of religious beliefs, when the eclipse of the sun occurred, the Saudi government postponed the activities happening in the two cities, while instructing the residents to pray in mosques. This is because the religion of Islam requires Muslims to pray until the eclipse ends. The government then provided the people, whose life was disrupted because of the cancellation of the city events, with accommodation and an apology. Through publishing these posts, the government ensured the residents that the two cities accepted cultural diversity and were good places for residents to live, visit and work in. Moreover, the government deployed the teachings of the holy Qur’an to boost the people’s pride of living in the Saudi cities with comfortable lifestyle. It asked the residents to thank Allah for all the benefits they received owning to the growth of the cities. Table 2 presents the result.

**Table 2:** City branding: Jeddah and Riyadh.

Dimensions		Jeddah City			Riyadh City			Total
		*Twitter*	*Facebook*	*Instagram*	*Twitter*	*Facebook*	*Instagram*	
*Physical attributes*	Cafe’s design	11	7	9	27	19	15	194 (%15)
	Religious architecture	13	2	7	20	2	10	
	Religious colour	2	2	3	3	4	3	
	Religious logos	2	1	1	15	2	3	
	Religious figures	5	0	1	4	0	1	
*Functional attributes*	Clean environment	12	14	8	19	8	6	666 (%53)
	Business opportunities	1	2	3	0	3	4	
	Cultural activities	107	41	52	181	67	87	
	Transport	19	5	3	6	5	5	
	Government Services	5	3	0	0	0	0	
*Personality traits*	Social bonding/community	14	17	19	27	25	30	393 (%32)
	City brand attitudes	32	9	8	11	48	85	
	Intentions	0	0	0	2	0	0	
	Religious beliefs	11	8	4	13	2	7	
	Religious values	3	0	0	6	4	2	
	Religious ritual	2	0	0	2	1	1	

RQ2 explored how the residents responded to the government’s attempt on social media. The result showed that Twitter was the leading social media networking site where most of the government-and-resident interactions took place, compared with those on Facebook and Instagram. This was evidenced by the high E indexes in each of the three dimensions across the two festivals–see Table 3. In addition, the interactions on Instagram were more active than those on Facebook. This suggested that Twitter serves as the main channel for the government and the residents to access and circulate festival-related information, followed by Instagram and Facebook.

**Table 3:** Resident engagement with the cities of Jeddah and Riyadh.

City	Social media platform	Number of followers	Dimensions	P index	V index	C index	E index
Jeddah	Twitter	139000	Physical attributes	1.5	0.3	0.2	2.0
			Functional attributes	2.0	0.6	0.1	2.7
			Personality traits	1.6	0.4	0.1	2.1
Riyadh		320000	Physical attributes	1.2	0.7	0.1	2.0
			Functional attributes	1.7	0.8	0.1	2.6
			Personality traits	1.5	0.6	0.1	2.2
Jeddah	Facebook	55000	Physical attributes	0.3	0.2	0.1	0.5
			Functional attributes	0.5	0.3	0.1	0.9
			Personality traits	0.4	0.2	0.1	0.6
Riyadh		84000	Physical attributes	0.2	0.2	0.1	0.5
			Functional attributes	0.6	0.2	0.1	0.9
			Personality traits	0.4	0.2	0.1	0.7
Jeddah	Instagram	129000	Physical attributes	0.8	0.4	0.2	1.4
			Functional attributes	1.6	0.1	0.3	2.0
			Personality traits	1.3	0.3	0.4	2.0
Riyadh		108000	Physical attributes	0.9	0.3	0.1	1.3
			Functional attributes	1.7	0.6	0.2	2.5
			Personality traits	1.2	0.2	0.1	1.5

Note: Popularity (P), Virality (V), Commitment (C), and Engagement (E).

Zooming in on the engagement activities on Twitter, we found the residents were engaged in the government-led conversations about the functional attributes of the two city brands, followed by the personality traits and the physical attributes. This was supported by the E indexes in the functional attributes (2.7 in the Jeddah Season and 2.6 in the Riyadh Season) and the personality traits (2.1 in the Jeddah Season; and 2.2 in the Riyadh Season). The scores of these E indexes were largely attributed to the values of the P and the V, as the C index values were low in both seasons. This result gave the indication that resident engagement with the government posts on Twitter took the mode of "like" and "share", with a lack of "comment".

Specifically, the residents were highly engaged with the posts involving the dimensions of functional attributes and personality traits. Regarding their engagement with the functional attributes of the two city brands, the residents interacted with the government posts that showed the opportunities of entertainment available in the cities, such as outdoor amusements and cultural events. Likewise, this happened to the government posts categorized in the personality dimension, which portrayed social bonding, religious values, and beliefs. However, the residents did not commit to commenting on these posts. The behaviour of "comment" is considered as a high-level engagement mode, compared to the low-level engagement modes of “like” and “share” on social media [63]. Thereby, the users preferred not to exchange their ideas about the messages aired by the local governments regarding the two seasons of Jeddah and Riyadh. From this aspect, the government’s circulation of the information about the two festivals did not help to evoke high-level engagement among the residents.

In fact, the resident engagement with both the Jeddah and the Riyadh Season through the three city branding dimensions—physical attributes, functional attributes, and personality traits–were very similar across the three social media networking sites, with the values of the P and the V higher than that of the C index. These results showed residents’ attitudes towards the Jeddah and the Riyadh Season. Therefore, topics relating to personality traits (e.g., social bonding, religious values, and beliefs) and those linking with functional attributes (e.g., cultural activities that enable residents to have a better life), contributed to active engagement between residents and the government on social media. However, the overall outcome of resident engagement with the two seasons of Jeddah and Riyadh on Twitter was better than that on Instagram and Facebook. Therefore, Instagram and especially Facebook were not the effective channels to enhance government-resident interactions on social media.

Table 3 outlines the engagement details.

## Discussion and conclusion

Religion serves as an important aspect in branding cities within Saudi Arabia. This study unpacked the role of religion in engaging stakeholders on social media in branding the two Saudi cities—Jeddah and Riyadh—and explored how this process mediated the meanings of the two city brands via applying content analysis.

### Discussion and contribution

The result of analyzing the narratives generated by the government and the residents showed that the Islamic religion serves as a powerful tool for the government to engage the residents in branding the two cities. The government capitalized on the religious factors (e.g., Islamic architecture, the Islamic Teachings, the Noble Qur’an, and The Farouq competition) to portray the two cities, with more attention given to the functional attributes and the personality traits than the physical attributes. This government effort spiked active response from the residents, evidenced by the high E index values in the two aforementioned dimensions. These findings reinforce the result of previous studies—stakeholder engagement with the personality traits of a place brand serves as a strategic approach to drive public participation in place branding initiatives [13], while allowing for fostering emotional connections and favourable relationships between stakeholders [18].

The result above extends the potential of religion in strengthening stakeholder engagement via delving into the functional attributions of a place brand. This is supported by the government positioning the two seasons as a religion-related cultural event to build resonance with the residents, which showed the alignment with tangible demand of the residents (e.g., better infrastructure and more cultural events). As noted, resident engagement with the government’s communications about the city functions (e.g., cultural activities, transport, and government services) creates a fertile ground for the generation of public trust towards the place and the government [63]. Thereby, religion plays a productive role in motivating the residents to engage in government-led city branding initiatives at the individual level. This role is comprehensive, blending the aspects of physical and functional attributes into personality traits of a place brand with a potential for contributing to inspiring social development.

Moreover, the residents engaged with the government through liking and sharing their posts–a low level of engagement. The lack of engagement at the high level represented by "comment" points to the residents’ concerns about exchanging personal views on government content in the digital space. This is likely to be attributed to the communication mechanism in Saudi Arabia. For instance, the Saudi government has imposed regulations on speech and expression over the dissemination of material that violates Islamic law, weakens national security, and advances the interests of foreigners [66,67]; and there are government attempts to monitor information on the internet [67]. This reasoning corresponds with the findings of previous research that power asymmetries in government-public relationships constrain the engagement of local communities (residents and citizens) in the communication practice of place branding; and stakeholder engagement driven by the government could serve as a strategic approach embedded in place branding exercises to enhance internal governance. From this aspect, the potential of utilizing religion to move resident engagement beyond the individual level towards the social level for the purpose of growing resources to facilitate social development within the Saudi context should be reassessed.

In the meantime, in comparison with Instagram and Facebook, Twitter was found to be the primary social network site where most of these engagement activities took place. This finding is in the alignment with the demographic features of the digital media landscape in Saudi Arabia, in which Twitter is the most popular platform and Twitter users account for the majority rather than users on the other social media networking sites [56].

Theoretically, the discussions about the contribution of religion to a city brand from the dimensions of physical attributes, functional attributes and personality traits expand the scope of place branding research. It points to the critical role of religion in driving resident engagement with government-led initiatives, while forging the connections between the government and the residents through the consensus on religious values. This as a result allows for achieving the goals of the government (e.g., economic growth and sustainable governance) as well as the residents (e.g., better quality of life) in the process of building a place brand. Linking the principles of stakeholder engagement with the three-dimensional essence of a place brand, this article puts forward the framework of religion city branding through stakeholder engagement. In line with this framework, the government should engage the residents in co-developing the three dimensions of a city brand—physical attributes, functional attributes and personality traits—at the individual level; and the success of this individual-level engagement in city branding initiatives creates the basis for generating the social-level engagement that helps the growth of the resources in support of social development in a religious society. This framework, therefore, underpins tangible and intangible rewards of city branding exercises through the lens of religion.

Practically, in the pursuit of the success of city branding within the religious setting, it is important for stakeholders to draw more attention to shaping the personality traits of a city brand via creating shared values and beliefs aligning with Islamic values. Arab residents are bound to embrace the mechanisms formed by the government to strengthen the status of religion in their countries [1]. The Islamic values of tolerance and justice are widespread in the Middle East and act as the guide for formulating and implementing policies in business and legal operations [1,68]. Accordingly, the incorporation of these Islamic values into the development of the three-dimensional meanings of a city brand would help to provoke resident engagement in city branding processes and contribute to positive relationships between the government and the residents. To implement this strategy, the government and pro-government actors should communicate ethically with residents on social media, especially on Twitter. For instance, take into account the reactions of users and offer feedback to their comments and queries. These responsive acts from the government would encourage users to like, share and post comments, eliciting more interactions between stakeholders on social media [69,70]. It in return would enlighten a higher level of engagement at both the individual and social levels.

The outcomes of this study add values to stakeholder engagement in place branding research by uncovering the ways religion mediates the three-dimensional meanings of a city brand. It presents insights into the attempts launched by the Saudi government through the reliance on religion to evoke a participatory response from the residents during the two seasons of Jeddah and Riyadh. Due to the increasing use of festivals by the states in the Middle Eastern countries to advance political aims through religion, this Saudi-focused study possesses domestic and regional references.

### Limitations and future research

As with any case study research, our study is constrained by the ability to generalise the findings beyond the context of Saudi Arabia to other countries. Future research centering on a bigger sample size within the same country and in different countries located in and outside the Middle Eastern setting would add weight to this research. Another limitation of this study is associated with the inclusion of the two religious festivals taking place in the two cities located in Saudi Arabia—Jeddah and Riyadh. Future studies looking at the city branding practices in the two cities happening in different periods outside the timeframe of these two festivals would present a better view of the role of religion in city branding. Moreover, our study focuses on examining social media data generated by the key stakeholders of the government and residents using the method of content analysis. Future researchers are recommended to probe engagement behaviors among other types of stakeholder groups, such as corporate stakeholders, social media influencers and NGO stakeholders, with the consideration of adopting the survey or the in-depth interview method [13]. This would allow the researchers and practitioners to better understand the engagement drivers of a wider range of stakeholder groups (e.g., motivations) in city branding initiatives through the lens of religion.
